# Editorial: Positive emotion dysregulation in eating disorders and dysregulated eating behaviors

**DOI:** 10.3389/fpsyg.2024.1437889

**Published:** 2024-06-26

**Authors:** Edward A. Selby, Lindsay P. Bodell, Ann F. Haynos

**Affiliations:** ^1^Department of Psychology, Rutgers, The State University of New Jersey, New Brunswick, NJ, United States; ^2^Department of Psychology, University of Western Ontario, London, ON, Canada; ^3^Department of Psychology, Virginia Commonwealth University, Richmond, VI, United States

**Keywords:** eating disorder, positive affect, excessive exercise, binge and purge, restricting

Positive emotions can play complex roles in eating disorders and dysregulated eating behaviors, influencing both the development and maintenance of these problems. However, research focused on the impact of positive emotions and their dysregulation in these conditions is notably lacking as compared to studies on negative emotion dysregulation. Positive emotions in these conditions should not be neglected, as a growing literature indicates that positive emotion dysregulation is implicated in various behaviors across the eating disorders spectrum, including overconsumption of palatable food, binge eating, restrictive eating, and excessive exercise (Selby et al., 2015; Coniglio et al., 2019). Positive emotional experience can also affect higher-order aspects of human functioning, such as happiness, life satisfaction, motivation, and fulfillment, all of which tend to be impaired in individuals with eating disorder symptoms.

Key questions in the field revolve around whether dysregulated behaviors stem from diminished positive emotions and whether eating behaviors are reinforced by maladaptive emotional responses. Integration of positive emotion dysregulation into our theoretical models can improve our understanding of the onset and maintenance of eating disorders and dysfunctional eating behaviors (Selby and Coniglio, 2020; Bodell and Racine, 2023). Likewise, novel treatments for eating disorders may involve addressing the interplay between positive emotions, negative emotions, and maladaptive eating behaviors to promote healthier coping strategies and emotional regulation skills. Therefore, the better we can understand the key roles of positive emotion, the more precisely we can specify our treatments (Haynos et al., 2016, 2024).

This Research Topic aims to continue these lines of work and draw attention to ongoing research on positive emotion dysregulation in problematic eating behaviors. Included articles examined the role of positive emotion in disordered eating through factors such as emotion regulation, emotional reactivity traits, attachment patterns, and the dynamic interplay between psychosocial factors and eating behaviors. Collectively, these findings highlight that although positive emotions can serve adaptive functions at times, they can also contribute to the complexity and maintenance of eating disorders and dysregulated eating when they become dysregulated or become intertwined with disordered eating, weight loss, or social perception patterns.

*Positive emotional reactivity and regulation*: Positive emotional experience can serve as a coping mechanism, helping individuals regulate negative emotional reactivity (Selby et al., 2019; Bodell et al., 2022). In individuals with eating disorders, however, positive emotions may be sought out to manage or escape from negative emotions in a maladaptive way. For example, binge eating may temporarily alleviate distress and increase positive feelings. In their study, Fischer et al. investigated the relationship between alcohol use and objective binge eating in emerging adults, considering affect regulation processes. They suggested that alcohol consumption, particularly in the context of positive mood and impulsivity, may lead to binge eating episodes. Using ecological momentary assessment with an undergraduate sample, their study revealed that alcohol use increased the likelihood of objective binge eating later in the day. Positive urgency, the tendency to act impulsively in positive emotional states, was also associated with increased odds of binge eating following alcohol use. Similarly, in their study, Davis and Smith investigated the association between dispositional traits related to emotion regulation and binge size in bulimia nervosa, specifically negative urgency, the tendency to act rashly in distress, and positive urgency. Data from a mixed sample of women with bulimia nervosa and control participants revealed that individuals with bulimia nervosa scored higher on negative urgency, positive urgency, and negative affect. Elevated positive urgency predicted significantly greater test meal intake – particularly for individuals with bulimia nervosa. Overall, these collective findings highlight the transdiagnostic nature of positive emotion dysregulation, which may facilitate several eating disordered behaviors, as well as a larger set of dysregulated behaviors.

*Reward and reinforcement*: Certain eating behaviors, such as restriction or overeating, may be reinforced by positive emotions through the brain's reward system. In their study, Flynn et al. examined the relationship between positive affect and binge eating frequency and size among treatment-seeking adults with recurrent binge eating. Participants completed surveys assessing positive and negative affect as well as the frequency of objective and subjective binge episodes over the past 3 months. Lower positive affect was found to be significantly associated with more frequent total binge episodes, but not with objective or subjective binge episodes individually. These important findings suggest that increasing positive affect through activities outside of eating disorder behavior may be an important consideration in the treatment of individuals with recurrent binge eating. In another example, Lv et al. explored the influence of conscious and unconscious emotional processing on the dietary behavior of restrictive dieters. Female participants were categorized into successful, unsuccessful restrictive, and unrestricted eaters and underwent emotional priming tasks to assess eating intentions. Results indicated that restrictive dieters showed increased willingness to eat when in a positive mood, even unconsciously; unsuccessful restrictive dieters demonstrated an even stronger effect. Thus, consuming palatable foods or achieving weight loss goals can trigger feelings of pleasure and satisfaction, reinforcing the behavior and contributing to its persistence. Future treatments should specifically address how to address and ameliorate this reinforcement process.

*Social reinforcement:* Positive emotions associated with social interactions, such as feeling accepted or admired by others, can also influence eating behaviors. For example, as previously noted in the study by Fischer et al., social drinking episodes were commonly connected to binge eating episodes, with participants often endorsing “to have fun” as a reason for drinking that led to binge eating. Individuals with eating disorders may also seek social approval or validation through their appearance or eating habits, leading to disordered eating behaviors to functionally maintain positive social connections. Roithmeier et al. added to this growing literature by investigating the relationship between primary emotion traits, attachment patterns, and eating disorder behaviors in a non-clinical sample. Results indicated that greater problematic eating behavior was associated with lower positive emotions and higher negative emotions and attachment anxiety. These findings highlight the nuanced association between emotional experience, attachment security, and eating disorder symptoms, with future work needed to examine what other ways positive emotion and attachment concerns may facilitate eating disorder symptoms.

## Future directions

The primary aim of this Research Topic was to encourage focused research on the role of positive emotion dysregulation in eating disorders and associated behaviors. The current set of studies should be viewed as an initial step forward; however, additional work is needed in the following areas. First, we need to continue to identify new and more nuanced processes of positive emotion dysregulation that influence maladaptive eating behaviors, thoughts, or self-conceptualization, including their impact on weight and shape concerns. Along these lines, there is extremely limited research on the role of positive affect in certain forms of eating pathology, such as avoidant/restrictive food intake disorder and atypical anorexia nervosa. Second, we should continue to explore empirical methodologies for investigating positive emotional responses among individuals with eating disorders or disordered eating behaviors in both laboratory and ecologically relevant settings. Third, ongoing experimental, conceptual, and intervention work should aim to elucidate the social dimensions of positive emotion dysregulation in the development and persistence of disordered eating patterns. Fourth, further treatment research is needed to investigate how to alter positive affect dysregulation in eating disorders and related behaviors. Finally, future work should explore the broader effects of positive emotions, such as motivation, life satisfaction, identity, and social connection, to gain a deeper understanding of the contextual factors surrounding disordered eating symptoms. Through such collective efforts, we will undoubtably advance knowledge and inform interventions for individuals affected by eating disorders and related behaviors.

## Author contributions

ES: Conceptualization, Writing – original draft, Writing – review & editing. LB: Conceptualization, Writing – original draft, Writing – review & editing. AH: Conceptualization, Writing – original draft, Writing – review & editing.
